# Achieving a normal life in hereditary angioedema: Quality of life and treatment gaps among German HAE patients 

**DOI:** 10.5414/ALX02603E

**Published:** 2026-02-24

**Authors:** Markus Magerl, Thomas Buttgereit, Inmaculada Martinez-Saguer, Petra Staubach-Renz, Jens Greve, Emel Aygören-Pürsün, Lucia Schauf, Kathrin Schön

**Affiliations:** 1 Institute of Allergology, Charité – Universitätsmedizin Berlin, Corporate Member of Freie Universität Berlin and Humboldt-Universität zu Berlin,; 2 Fraunhofer Institute for Translational Medicine and Pharmacology ITMP, Immunology and Allergology, Berlin,; 3 HZRM Haemophilia Center Rhein Main, Frankfurt am Main,; 4 University Medical Center, Mainz,; 5 Department of Otorhinolaryngology, Head and Neck Surgery, Ulm University Medical Center, Ulm,; 6 University Hospital Frankfurt, Department for Children and Adolescents, Frankfurt, and; 7 HAE Vereinigung e.V., Germany; *Authors contributed equally; Prof. Marcus Maurer made invaluable contributions to this assessment. His expertise and insights were instrumental in shaping the project. We dedicate this work to his memory, honoring his legacy in advancing the understanding of hereditary angioedema.

**Keywords:** hereditary angioedema, quality of life, long-term prophylaxis, on-demand therapy, attack frequency, emotional impact, social engagement, patient-reported outcomes, HAE guidelines

## Abstract

Background: Hereditary angioedema (HAE) is a rare genetic disease characterized by recurrent swelling attacks. Current guidelines for HAE management emphasize achieving complete disease control to normalize patients’ lives. Quality of life (QoL) differences between patients with 0 attacks and those with persistent attacks remain to be explored. Materials and methods: The German patient organization for individuals affected by hereditary angioedema, HAE Vereinigung e.V. conducted an online survey with 122 HAE patients in Germany in 2024. Participants were categorized according to their therapy – long-term prophylaxis (LTP) or on-demand therapy (ODT) – and according to their attack frequency over the last 6 months. Patient-reported outcomes for functional, emotional, and social impacts were analyzed to evaluate QoL. Results: Although 83% of patients expressed satisfaction with their treatment, 59% of patients still had attacks. Patients on LTP reported significantly fewer attacks (p < 0.001) and higher QoL compared to those on ODT (p < 0.001). Patients with 0 attacks consistently showed significantly better outcomes across all QoL domains than those with 1 or more attacks (p < 0.001). Conclusion: The findings highlight that even minimal residual disease activity can meaningfully reduce QoL. Achieving complete attack freedom, rather than partial control, is necessary to restore normalcy for patients with HAE. Hence, regular adjustments of the HAE management plans based on patient-reported outcomes are crucial to ensure that treatment strategies address both medical and QoL needs.

## Introduction 

Hereditary angioedema (HAE) is a rare genetic disorder marked by recurrent swelling episodes affecting skin and mucous membranes [1, 2]. Symptoms often start in childhood and persist lifelong [1]. Estimated prevalence ranges from 1 : 50,000 to 1 : 100,000 [3]. 

HAE causes painful swelling, often in the face, limbs, and mucosa of the gut or airways. Respiratory involvement can be life-threatening [4, 5, 6]. As attacks are unpredictable, treatment is either on-demand or prophylactically [2]. The impact of the disease on quality of life (QoL) extends beyond severity and frequency of attacks. HAE patients frequently experience anxiety or depression and are more prone to calling in sick or being hospitalized during an attack [5, 7]. In addition to decreased productivity during and between attacks, HAE also hinders patients from advancing their career or forces them to adapt their life to the disease [5, 7]. 

In the context of long-term HAE management, the overarching objective is to attain complete disease control, thereby enabling patients to lead “normal” lives [2]. Effective control improves QoL by reducing the frequency and severity of attacks, as well as the associated emotional distress [8]. Despite progress, assessing real-world effectiveness in achieving these goals is essential [9]. 

The aim of this survey was to identify differences in QoL between patients with and without complete attack control in a German cohort by examining the functional, emotional, and social impacts of varying attack frequencies. 

## Materials and methods 

### Survey design and participants 

This cross-sectional survey was conducted online. The questionnaire was developed by the German association HAE Vereinigung e.V., supported by HAE experts, and was refined through a pilot survey prior to full distribution.

Participants (adults ≥ 18 years with HAE or guardians of affected minors) were recruited via the HAE Vereinigung e.V. website, social media, and expert-center referrals. Confirmation of diagnosis and treatment was ensured through self-reporting of the treating specialist center. 

A total of 122 participants completed the quantitative portion of the survey. Given the estimated prevalence of HAE (1 : 50,000 – 1 : 100,000), this sample size is considered substantial for rare-disease research and sufficient for meaningful subgroup analysis. 

The majority of respondents (n = 92, 75%) also responded to the qualitative research questions. Full-survey participants received €50; partial participants €25. All participants gave informed consent, and the survey was anonymous and did not collect sensitive health data. 

### Survey content 

In this patient survey, a recall period of 6 months was employed. The survey relied on self-reported diagnosis, treatment, and outcomes. While this introduces potential for recall bias, it captures real-world perceptions of disease control – our primary focus. A combination of quantitative and qualitative methodologies was used to assess the HAE patient experience. The assessment was divided into four sections. 

**QoL with HAE**

The questionnaire covered key areas of disease impact, using single-choice questions with a 5-point Likert scale (1 = Not at all, 2 = Rarely, 3 = Occasionally, 4 = Often, 5 = Very often) (Supplemental material, Appendix 1.). 

**Self-reflection**

Participants chose 1 image from 8 photographs showing emotional states or lived experiences related to HAE (Supplemental material, Appendix 2). This image set was developed and validated by HAE Vereinigung e.V. and the research team. 

**Knowledge sharing**

Participants shared individual coping strategies, contributing experiential knowledge to benefit the broader HAE patient community. 

**Life changes**

The participants submitted images representing life changes since starting therapy and described them in open text. 

### Statistical analysis 

IBM SPSS software, version 30.0.0.0 (172), was used to conduct quantitative analyses. One-way analysis of variance (ANOVA) was used to evaluate disparities in QoL across three categories of attack frequency (0 attacks, 1 – 3 attacks, > 3 attacks) within the previous 6 months. F-values from one-way ANOVA were reported to identify variations in QoL. Independent t-tests compared overall QoL scores between patients on long-term prophylaxis (LTP) and those using on-demand therapy (ODT). Comparisons of QoL between treatment types were not conducted within attack categories. Significance was set at p < 0.05. 

The publication focuses primarily on quantitative data analysis, but the answers to qualitative questions were used to enhance contextual comprehension. Selected open-text responses were grouped thematically and referenced to illustrate patient perspectives (Supplemental material, Appendix 3). 

## Results 

### One in three patients was symptom-free at the time of survey 

The survey, conducted in May-June 2024, included 122 HAE patients, with 68 (56%) using LTP and 54 (44%) ODT. Most participants had HAE-C1INH type I (72.1%) and were between 26 and 35 (26.2%) years old. Age was categorized; continuous age data, gender, and ethnicity were not collected (Table 1). 

### Perceived stability masks ongoing attack burden 

Although 83% of patients felt their HAE was well controlled, only 31% reported being attack-free. Attack frequency in the past 6 months varied:

- 38 patients (31%) experienced 0 attacks.
- 34 patients (28%) experienced 1 – 3 attacks.
- 50 patients (41%) experienced > 3 attacks.

**Figure 1:**
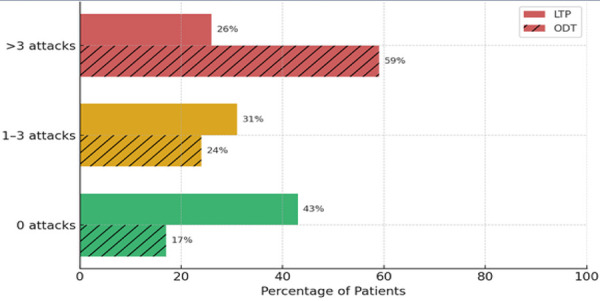
Comparison of attack frequency in the past 6 months of patients with hereditary angioedema on LTP vs. ODT. LTP = long-term prophylaxis; ODT = on-demand therapy.

Among LTP patients, 43% (n = 29) had 0 attacks, compared to only 17% (n = 9) of the ODT group, indicating a significant reduction in attack frequency associated with LTP (F(2, 119) = 149.35, p < 0.001). While a comparable proportion of patients using LTP or ODT reported 1 – 3 attacks (31 vs. 24%, respectively), a greater proportion of ODT users experienced > 3 attacks (59 vs. 26%) (Figure 1). 

### Even low numbers of attacks can have a significant impact on QoL 

**Figure 2:**
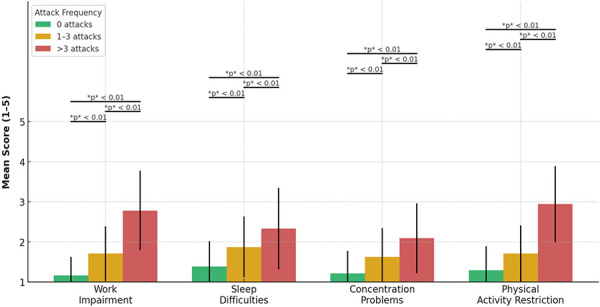
Functional impact by attack frequency in the past 6 months.

The functional burden of HAE increased with attack frequency across all assessed outcomes (Figure 2). In particular, the analysis revealed that an attack-free status leads to a significantly higher QoL even compared to those who experienced 1 – 3 attacks in the past 6 months. Patients with 1 – 3 attacks reported significantly greater physical limitations (p < 0.001), higher emotional burden (p < 0.001), and more frequent cancellations of social activities (p < 0.001) compared to those without any attacks. 

The degree of work impairment was lowest among attack-free patients (mean = 1.16, SD = 0.46), intermediate in those with 1 – 3 attacks (mean = 1.71, SD = 0.68), and highest in the > 3 group (mean = 2.78, SD = 0.99). 

A similar pattern was observed for sleep difficulties (mean = 1.38, SD = 0.64; mean = 1.87, SD = 0.76; mean = 2.33, SD = 1.02), F(2,119) = 20.38, p < 0.001, and for concentration problems (mean = 1.21, SD = 0.56; mean = 1.62, SD = 0.72; mean = 2.09, SD = 0.87), F(2,119) = 21.94, p < 0.001. 

The evaluation of physical activity restriction demonstrated a similar trend. Participants without attacks reported the lowest burden (mean = 1.29, SD = 0.60), followed by the 1 – 3 attacks group (mean = 1.71, SD = 0.70), and those with > 3 attacks (mean = 2.94, SD = 0.95); F(2,119) = 45.83, p < 0.001. 

These results underscore a consistent and significant functional impact of HAE that intensifies with increasing attack frequency, particularly in domains critical to daily work, rest, and physical mobility. 

The modality of treatment appeared to contribute to differences in reported functioning. Among patients with more than 3 attacks, those using only ODT reported more severe impairment in their work or daily lives (17%) compared to those on LTP (10%). Open-text responses in this group often referenced missed obligations and difficulty maintaining stable routines. 

Even among attack-free patients, a small subgroup on ODT (n = 9) indicated slightly higher scores for work impairment, sleep difficulties, and concentration problems than those on LTP (n = 29). No statistical comparisons were made due to the small sample size. 

### Emotional burden is greater among patients facing breakthrough attacks 

**Figure 3:**
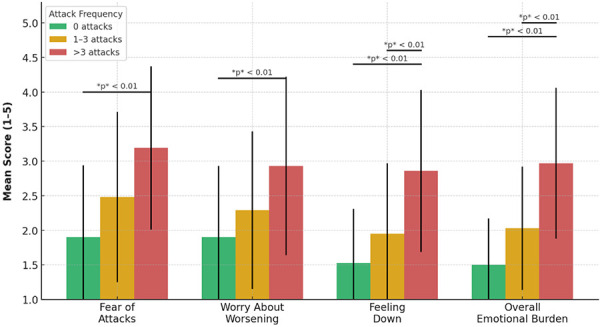
Emotional impact by attack frequency in the past 6 months. Mean scores and standard deviations (SD) for emotional burden by attack frequency category: 0 attacks (green), 1 – 3 attacks (orange), and > 3 attacks (red). Significant differences between groups are indicated by horizontal bars with corresponding p-values. Scores based on a 5-point Likert scale ranging from 1 (not at all) to 5 (very often).

The emotional burden of HAE increased significantly with attack frequency. Those who had no attacks in the past 6 months scored lower on emotional burden items than those with more frequent attacks. Figure 3 shows the mean scores and standard deviations for each group. 

The fear of unexpected attacks was lowest in the attack-free group (mean = 1.90, SD = 1.04), moderate in the 1 – 3 attacks group (mean = 2.48, SD = 1.23), and highest in the > 3 attacks group (mean = 3.19, SD = 1.18); F(2,119) = 16.14, p < 0.001. Subsequent analyses confirmed significant differences between the groups (p < 0.01). 

A similar pattern was observed for overall emotional burden (mean = 1.50, SD = 0.67; mean = 2.03, SD = 0.89; mean = 2.97, SD = 1.09); F(2,119) = 33.22, p < 0.001. Additional post hoc tests confirmed significant differences (p < 0.01). 

Also, the overall difference regarding the concern about worsening of symptoms (mean = 1.90, SD = 1.03; mean = 2.29, SD = 1.14; mean = 2.93, SD = 1.29) was significant, but the contrast between the 0 and 1 – 3 attack groups did not reach statistical significance. 

A similar pattern was found for feelings of being down (mean = 1.53, SD = 0.78; mean = 1.95, SD = 1.02; mean = 2.86, SD = 1.17); F(2,119) = 24.90, p < 0.001. In this case as well, post hoc analysis only revealed statistically significant differences between the > 3 attack group and the other two groups. 

Qualitative statements from participants underscored relief and restored emotional stability reported by attack-free participants (Supplemental material, Appendix 3). 

Subgroup analysis by treatment modality revealed that emotional burden was more frequently reported among ODT patients, particularly in the >3 attacks group. For instance, 61% of ODT users in this group reported frequent fear of attacks, compared to 36% of LTP users. These patterns suggest a relevant context for interpreting burden by treatment type. 

### Social confidence is strongest among patients living without attacks 

Social burden varied across attack frequency categories. Those free of attacks for at least 6 months showed the least restrictive patterns, particularly in domains pertaining to event cancellations and visibility avoidance. 

Attack frequency was positively correlated with the occurrence of event cancellations. Patients without attacks reported the lowest burden (mean = 1.08, SD = 0.57), followed by those who experienced 1 – 3 attacks (mean = 1.44, SD = 0.62) and those who experienced > 3 attacks (mean = 2.36, SD = 0.94). This difference was statistically significant (F(2,119) = 27.86, p < 0.001). Post hoc analysis confirmed significant differences among all groups (p < 0.01). 

The gradient was steepest for avoidance of visibility: the mean was 1.59 (standard deviation (SD) = 0.64) in the 0 attacks group, 1.76 (SD = 0.73) in the 1 – 3 attacks group, and 2.94 (SD = 1.17) in the > 3 attacks group. The F-statistic (F) was 32.78, with p < 0.001. Post hoc analysis showed significant differences between all three groups. 

Furthermore, more attacks were linked to less leisure time (mean = 1.58, SD = 0.68; mean = 1.62, SD = 0.74; mean = 2.86, SD = 1.15); F(2,119) = 22.39, p < 0.001. The difference between the 0 and 1 – 3 groups was not statistically significant. 

Feelings of “shame in public” followed a comparable pattern (mean = 1.11, SD = 0.59; mean = 1.29, SD = 0.66; mean = 2.12, SD = 0.85); F(2,119) = 10.21, p < 0.001. In this case as well, only comparisons involving the > 3 attacks group were statistically significant. 

**Figure 4:**
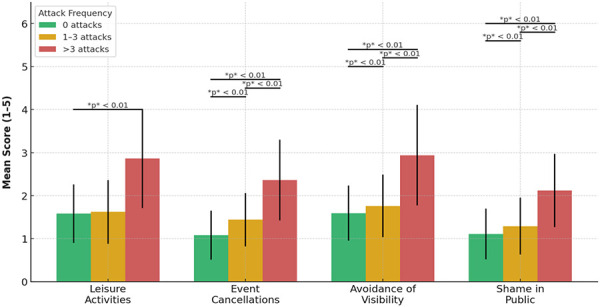
Social impact by attack frequency in the past 6 months.

Figure 4 presents group-wise means and standard deviations for all social impact items. 

The participants’ qualitative responses align with the quantitative findings, showing restored confidence, freedom, and social spontaneity in situations where visible attacks are no longer anticipated (Supplemental material, Appendix 3). 

Descriptive comparison suggested a higher burden among ODT users experiencing > 3 attacks in 6 months. For instance, 44% of ODT users reported frequent avoidance of visibility, compared to 24% of those on LTP. No statistical comparisons were conducted between treatment types within the groups, but the data suggest that ODT patients may perceive greater social limitation during frequent attacks. 

## Discussion 

This survey provides clear evidence of the importance of being attack-free for QoL of patients with HAE. 

### The majority feel in control – but only a minority is attack-free 

While 83% of patients reported satisfaction with their current treatment, only 31% reported being attack-free in the past 6 months. Of those with breakthrough attacks, 28% had 1 – 3 attacks, while 41% had > 3 attacks in the past 6 months. This discrepancy underscores a fundamental divergence between patients’ perceptions of stability and their actual level of attack control. Of particular significance is the observation that only 44% of patients under LTP were attack-free while 26% experienced > 3 attacks in the past 6 months. The absence of severity data paradoxically strengthens the relevance of our findings: based on data of clinical trials we expect the breakthrough attacks to be less frequent and less severe [22, 23, 24]; this makes the disproportionate impact on QoL even more striking. This underscores that the threshold for “complete disease control” needs to shift toward attack freedom, particularly when aiming to normalize QoL [25]. 

Patients who were attack-free consistently scored best across all QoL domains. They reported minimal disruption to their daily routines, reduced emotional burden, and higher confidence levels when engaging in social interactions. Patients with attacks reported more limitations in their work, education, and emotional well-being. This makes it clear that achieving an attack-free status should be regarded as a meaningful marker of treatment success, as opposed to reliance on subjective impressions of disease control. This underscores the necessity for personalized, attack-free HAE management strategies, as recommended in the current guidelines [2]. 

The observed potential for enhanced QoL under prophylaxis is further substantiated by the flexibility inherent in HAE management. Unlike many other chronic conditions, therapeutic decisions for HAE can be continuously adapted to changes in patients’ lives. This distinctive adaptability can facilitate the initiation of prophylactic therapy, providing both physicians and patients the assurance that treatments can be optimized and adjusted at any point to meet evolving individual needs. Further research with sufficient samples is needed to explore how treatment strategy influences QoL, even in patients who no longer experience attacks. 

### Turning the ultimate HAE treatment goals into reality 

Despite the efficacy of LTP in reducing attacks, achieving an attack-free life remains challenging for many. In this survey, 43% of patients using LTP reported no attacks within the past 6 months, and 26% still experienced more than 3 attacks. These findings align with clinical trials, which also show ~ 30% of LTP patients continue to experience breakthrough symptoms despite treatment [6, 9]. 

This discrepancy highlights a key observation: current therapies may satisfy many patients but do not guarantee an attack-free state linked to optimal QoL outcomes. Attack-free patients in this survey reported lower emotional burden, stronger functional capacity, and greater confidence in social interactions – benefits that define a “normal life”. 

While this analysis focused on attack frequency, the differences between treatment modalities within the attack-free group may be important. Among patients who lived attack-free in the past 6 months, those receiving LTP had lower burden scores in both functional and emotional domains compared to those using ODT. For example, mean scores for work impairment, concentration difficulties, and fear of attacks were lower in LTP users. Despite the absence of statistical testing due to minimal subgroup sizes within the attack-free group (n = 38), these comparisons imply that the quality of attack freedom may differ depending on treatment modality, potentially offering greater stability and reassurance and achieving a different level of normalization of life under prophylaxis. These findings call for further research to explore whether LTP enables a deeper level of normalcy even among patients with the same attack frequency. 

Prior studies [3, 6] have described the emotional and functional burden of breakthrough attacks, yet few have isolated the distinct impact of achieving 0 attacks. Innovation is key to address the gap between the perceived level of control and the actual alleviation of attacks. Recent therapeutic advancements hold the potential for improved attack control, reduced burden of treatment, greater convenience, and enhanced tolerability [10]. Addressing the discrepancy between treatment satisfaction and the goal of an attack-free life necessitates a more structured, patient-centered approach. The 3D model of shared decision-making – discover, discuss, decide – provides a practical framework to align clinical choices with patients’ lived experiences and long-term aspirations [11]. 

The integration of new treatment options and shared decision-making into routine care can help patients move beyond a state of merely “doing well” to one of living without restrictions in all areas of life. 

### Limitations 

This survey has several limitations. First, the sample was restricted to patients receiving treatment in Germany. Although findings may not be generalizable beyond Germany, the focus on one national system ensured consistency in treatment access, making observed differences more likely attributable to clinical variables than to disparities in access to particular treatment options [26]. 

Second, this retrospective survey design relied on self-reported diagnosis, treatment, and outcomes which introduces a potential for recall bias; in addition, no data concerning the severity of the attacks was gathered, which limits our ability to distinguish between mild and severe breakthrough events. Furthermore, treatment modalities were consolidated into a unified category, without supplementary differentiation or information concerning treatment duration. 

Third, the demographic data collected was limited, as it did not include information on gender and ethnic background. Finally, the survey did not use standardized disease control instruments (e.g., angioedema control test [12]). Instead, it used verbal frequency scales (e.g., “very often” to “not at all”) for patient self-assessments. This format is easy to use and captures perceived burden but limits the comparability of results. 

## Conclusion 

Despite current prophylactic therapies in the management of HAE, the results of this survey emphasize persistent challenges faced by many patients in achieving an attack-free life. This disconnection underscores the incongruity between perceived management of the disease and potential enhancements of QoL. 

Attack-free patients reported enhanced functional performance, emotional stability, and social participation. These findings suggest that freedom from attacks may represent the most direct path to a normal, unrestricted life. However, a considerable proportion of patients still have breakthrough attacks despite ongoing LTP. 

Regular, outcome-based reassessment of patients, as well as personalized therapy plans, remain essential to ensure treatment success matches patients’ experiences and goals. New treatment options and treatments that are currently under development [13] offer promising options to further reduce attack frequency and treatment burden. 

Consequently, future research should explore how best to integrate these innovations into care models that emphasize both clinical effectiveness and QoL outcomes. 

## Acknowledgments 

The authors thank all patients and the HAE Vereinigung e.V. for their engagement and support in making this survey possible. We also acknowledge hvl360º for editorial assistance and medical writing support. CSL Behring financially supported the HAE Vereinigung e.V. to enable the implementation of this survey. We thank Beate Schinzel for her support with the submission of the manuscript. 

## Authors’ contributions 

T. Buttgereit and J. Greve led the conceptualization and writing of this manuscript. All authors contributed to the design and refinement of the survey concept. T. Buttgereit and J. Greve also coordinated the interpretation of results and manuscript review process. All authors provided critical feedback on manuscript drafts and approved the final version for submission. 

## Funding 

CSL Behring provided an independent grant to the HAE Vereinigung e.V. to support the implementation of this survey. The project was initiated, led, and carried out by the association. 

## Conflict of interest 

M. Magerl is or recently was a speaker and/or advisor for and/or has received research funding from BioCryst, Pharming, Takeda, CSL Behring, Intellia, Ionis Pharmaceuticals/ Otsuka, KalVista, and Pharvaris. T. Buttgereit is or recently was a speaker and/or advisor for Almirall, Aquestive, AstraZeneca, Biocryst, CSL-Behring, Galderma, GlaxoSmithKline, Hexal, KalVista, Medac, Novartis, Pharming, Pharvaris, Otsuka, Roche, Sanofi, Swixx BioPharma and Takeda. I. Martinez-Saguer is or recently was a speaker and/or advisor for and/or has received research funding from BioCryst, CSL Behring, KalVista, Pharming, Pharvaris, Octapharma, and Takeda. P. Staubach-Renz is or recently was speaker and/or advisor for BioCryst, CSL Behring, KalVista, Octapharma, and Takeda. J. Greve has received speaker/consultancy fees from CSL Behring, Kalvista, Otsuka, Takeda and BioCryst; he has also received funding to attend conferences/educational events from CSL Behring, Biocryst, and; he has participated as an investigator in a clinical trial/registry for CSL Behring, Pharvaris, BioCryst, and Takeda. E. Aygören-Pürsün has received grants from CSL Behring and Takeda and consulting fees from Astria, CSL Behring, BioCryst, Intellia, KalVista, Pharvaris, and Takeda (honoraria personal or to the institution); she has received speaker fees from CSL Behring, BioCryst, Centogene, Pharming, and Takeda, and has served as an advisor for Astria, BioCryst, BioMarin, Centogene, CSL Behring, Intellia, KalVista, Pharming, Pharvaris, and Takeda. L. Schauf declares no relevant conflicts of interest in relation to this work. K. Schön is or recently was speaker and/or advisor for BioCryst, CSL Behring, and Takeda. 

**Table 1.** Patient characteristics.

**HAE subtype**	**LTP (n = 68)**	**ODT (n = 54)**	**Total (n = 122)**
HAE-C1INH type I	52 (76.5%)	36 (66.7%)	88 (72.1%)
HAE-C1INH type II	5 (7.4%)	2 (3.7%)	7 (5.7%)
HAE-nC1INH	7 (10.3%)	4 (7.4%)	11 (9.0%)
HAE type unknown	4 (5.9%)	12 (22.2%)	16 (13.1%)
**Age group (years)**	**LTP (n = 68)**	**ODT (n = 54)**	**Total (n = 122)**
< 18	1 (1.5%)	6 (11.1%)	7 (5.7%)
18 – 25	8 (11.8%)	6 (11.1%)	14 (11.5%)
26 – 35	15 (22.1%)	17 (31.5%)	32 (26.2%)
36 – 45	9 (13.2%)	5 (9.3%)	14 (11.5%)
46 – 55	15 (22.1%)	10 (18.5%)	25 (20.5%)
56 – 65	16 (23.5%)	7 (13.0%)	23 (18.9%)
65	4 (5.9%)	3 (5.6%)	7 (5.7%)

LTP = long-term prophylaxis; ODT = on-demand therapy; HAE-C1INH = hereditary angioedema with C1 inhibitor deficiency; HAE-nC1INH = hereditary angioedema with normal C1 inhibitor. Percentages are within-treatment group totals.
